# Inhibition of dynamin-related protein 1 protects against myocardial ischemia–reperfusion injury in diabetic mice

**DOI:** 10.1186/s12933-017-0501-2

**Published:** 2017-02-07

**Authors:** Mingge Ding, Qianqian Dong, Zhenghua Liu, Zheng Liu, Yinxian Qu, Xing Li, Cong Huo, Xin Jia, Feng Fu, Xiaoming Wang

**Affiliations:** 1 Department of Geriatrics, Xi’an Central Hospital, Xi’an, 710003 China; 2 Department of Geriatrics, Xijing Hospital, Fourth Military Medical University, 15 Changlexi Road, Xi’an, 710032 China; 3 Department of Natural Medicine, School of Pharmacy, Fourth Military Medical University, Xi’an, 710032 China; 4 Department of Physiology, Fourth Military Medical University, 169 Changlexi Road, Xi’an, 710032 China

**Keywords:** Drp1, Mitochondrial fission, Diabetes, Ischemia–reperfusion

## Abstract

**Background:**

Many cardioprotective pharmacological agents failed to exert their protective effects in diabetic hearts subjected to myocardial ischemia/reperfusion (MI/R). Identify the molecular basis linking diabetes with MI/R injury is scientifically important and may provide effective therapeutic approaches. Dynamin-related protein 1 (Drp1)-mediated mitochondrial fission plays an important role in MI/R injury under non-diabetic conditions. Importantly, recent studies indicated that Drp1-mediated mitochondrial fission is enhanced in the myocardium of diabetic mice. The above evidences suggested that Drp1 may be one critical molecule linking diabetes with MI/R injury. We hypothesized that inhibition of Drp1 may be effective to reduce MI/R injury in diabetic hearts.

**Methods:**

High-fat diet and streptozotocin-induced diabetic mice were subjected to MI/R or sham operation. Mdivi-1 (1.2 mg/kg), a small molecule inhibitor of Drp1 or vehicle was administrated 15 min before the onset of reperfusion. Outcome measures included mitochondrial morphology, mitochondrial function, myocardial injury, cardiac function and oxidative stress.

**Results:**

Mitochondrial fission was significantly increased following MI/R as evidenced by enhanced translocation of Drp1 to mitochondria and decreased mitochondrial size. Delivery of Mdivi-1 into diabetic mice markedly inhibited Drp1 translocation to the mitochondria and reduced mitochondrial fission following MI/R. Inhibition of Drp1 in diabetic hearts improved mitochondrial function and cardiac function following MI/R. Moreover, inhibition of Drp1 reduced myocardial infarct size and serum cardiac troponin I and lactate dehydrogenase activities. These cardioprotective effects were associated with decreased cardiomyocyte apoptosis and malondialdehyde production and increased activities of antioxidant enzyme manganese superoxide dismutase.

**Conclusions:**

Pharmacological inhibition of Drp1 prevents mitochondrial fission and reduces MI/R injury in diabetic mice. The findings suggest Drp1 may be a potential novel therapeutic target for diabetic cardiac complications.

## Background

It has been widely recognized that cardiovascular disease is the primary cause of death among diabetic subjects. Patients with diabetes face an increased risk of morbidity from ischemic heart disease than non-diabetic population, with exacerbated myocardial injury and cardiac dysfunction following ischemia/reperfusion (I/R) [1–3]. Moreover, ischemic precondition and many cardioprotective pharmacological agents failed to exert their protective effects in diabetic ischemic hearts [4–6], which means many interventions that are effective in reducing myocardial ischemia/reperfusion (MI/R) injury are ineffective under diabetic condition. Therefore, identify the molecular basis linking diabetes with MI/R injury is scientifically important and may provide effective therapeutic approaches. As mitochondria have been considered as vital cell organelles in cardiomyocytes due to their pivotal role in ATP production required for myocardial contraction and survival, targeting mitochondrial pathological alterations may be one promising strategy against diabetic ischemic injury [7–9].

Recent studies indicate that mitochondria are dynamic organelles that undergo fission and fusion events, which serve to distribute mitochondria to daughter cells, to remove damaged mitochondria, and to help modulate cellular response to stress [10, 11]. Increased mitochondrial fission in the I/R heart has been demonstrated to contribute to reactive oxygen species (ROS) production and infarct generation, while inhibiting mitochondrial fission reduces myocardial injury and improves cardiac function following I/R [12, 13]. Dynamin-related protein 1 (Drp1) is a cytosolic protein that is recruited over the mitochondrial surface to initiate fission through interaction with binding partners as fission protein 1 (Fis1) [14]. Drp1 is translocated to mitochondria in models of myocardial I/R, resulting in mitochondrial fission and dysfunction. Inhibition of Drp1 has been demonstrated to reduce mitochondrial fission and infarct size during I/R injury in non-diabetic animals [13, 15]. These studies suggest that Drp1-mediated mitochondrial fission plays an important role in myocardial ischemia/reperfusion (MI/R) injury under non-diabetic conditions.

Diabetes has a direct effect on mitochondrial dynamics. Mitochondria become shorter and smaller under acute hyperglycemic conditions in a rapid response, which is also mediated by the fission protein Drp1 [16]. Increased mitochondrial fission associated with activated Drp1 is observed in coronary endothelial cells or cardiac myocytes from murine diabetic models [17, 18]. These studies indicate that Drp1-mediated mitochondrial fission is enhanced in diabetic hearts. The above evidences suggested that Drp1 may be one critical molecule linking diabetes with MI/R injury. Then, we hypothesized that inhibiting Drp1-mediated mitochondrial fission may reduce MI/R injury and improve cardiac function under diabetic conditions. The aims of this study were (1) to investigate whether an inhibitor of Drp1 was effective in reducing MI/R injury in diabetic animals, and if so, (2) further explore the underlying mechanisms.

## Methods

### Induction of type 2 diabetic mice

All animal experiments were performed in compliance with the National Institutes of Health Guidelines on Animal Research. All experimental procedures were approved by the Fourth Military Medical University Ethics Committee. The high-fat diet (HFD) and streptozotocin (STZ)-induced diabetic mouse model was developed according to previous studies from our lab and others [19, 20]. Male C57BL/6 J mice (8–10 weeks old) were fed with HFD (D12492, Research Diets) containing 60% fat (kcal%), 20% protein, and 20% carbohydrate for 4 weeks and then injected with a single low dose STZ (90 mg/kg, Sigma, St. Louis, MO, USA) intraperitoneally. One week after STZ injection, mice were considered to have developed diabetes and used for the study only if their 12-h fasting blood glucose levels were higher than 11.1 mmol/L. The successful rate of diabetic model was 91.4% (64 of 70 total mice). Fasting blood samples were collected to analyze serum TG (triacylglycerol) and insulin levels. The level of serum TG was analyzed using an automatic biochemical analyzer (Advia 2400, Siemens, Germany). Serum insulin level was determined by using an enzyme linked immunosorbent assay.

### Myocardial ischemia–reperfusion model

**Fig. 1:**
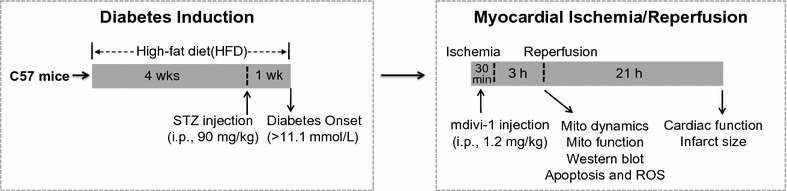
Schematic representation of experimental protocol. Male C57 mice were fed with HFD for 4 weeks and then injected with streptozotocin (STZ) at the dose of 90 mg/kg intraperitoneally. One week after STZ injection, mice were considered to have developed diabetes only if their 12-h fasting blood glucose levels were higher than 11.1 mmol/L. The diabetic mice were subjected to 30 min ischemia followed by 3 h reperfusion (for determination of mitochondrial dynamics, mitochondrial function, myocardial apoptosis and ROS) or 24 h reperfusion (for determination of infarct size and cardiac function). Mdivi-1 (1.2 mg/kg) or vehicle (dimethyl sulfoxide) was administrated intraperitoneally 15 min before the onset of myocardial reperfusion

Mice were anesthetized by intraperitoneal injection of 1% pentobarbital sodium. Myocardial ischemia was performed by exteriorizing the heart followed by a slipknot at the distal 1/3 of the left anterior-descending (LAD) coronary artery origin with a 6-0 silk suture. The slipknot was released after occlusion for 30 min, and the myocardium was reperfused for 3 h (for determination of mitochondrial morphology and apoptosis) and 24 h (for measurement of infarct size and cardiac function) as illustrated in Fig. 1. The mice were placed in a sterile environment and received penicillin treatment (5 unit/mice) to prevent infection at 3 h post-reperfusion. In sham-operated mice, the silk suture placed underneath the LAD artery was not ligated.

Diabetic mice were randomly assigned to receive either mdivi-1 (1.2 mg/kg), a small molecule inhibitor of Drp1 or vehicle (dimethyl sulfoxide) 15 min before the onset of myocardial reperfusion by intraperitoneal injection. The dose and route of mdivi-1 administration were chosen based on previous studies [15, 21, 22] and validated in our preliminary experiments.

### Subcellular fractionation and western blotting analysis

Whole-cell lysates and mitochondrial fractions were obtained from mice hearts in different groups. Whole cell lysates were prepared as previously described [12]. Mitochondrial fractions were prepared by using the Mitochondria Isolation Kit (Beyotime Biotechnology, Jiangsu, China). Western blot analysis was performed as previously described [23]. The following primary antibodies were used: anti-Drp1 (Abcam), anti-VDAC1 (Abcam), and anti-GAPDH (Wuhan Boster Biological Technology).

### Transmission electron microscopy

Ventricular samples were obtained from the proximal territory to artery occlusion site after 3 h of reperfusion. Samples were fixed in 2.5% glutaraldehyde in 0.1 M sodium cacodylate buffer (pH 7.2) overnight at 4 °C and processed as described before [24]. Images were collected using a JEM-1230 transmission electron microscope (JEOL Ltd., Tokyo, Japan) at 300 kV with a charge-coupled device (CCD) camera. Mitochondrial morphology was analyzed by using Image-Pro Plus software. For each heart, a minimum of 600 mitochondria was subjected to morphometric analysis. The percentage of mitochondria that fell into three size categories (smaller than 0.6 μm^2^, within 0.6–1.0 μm^2^, larger than 1.0 μm^2^) was analyzed in a given field according to previous studies [21, 25].

### Determination of mitochondrial function

Mitochondrial electron transport chain complex (I–IV) activities were measured by using specific commercial kits (Shanghai GENMED Technology Co., LTD, China) according to the standard protocols. Intracellular ATP content was determined using ATP bioluminescent assay kits (Biovision) according to manufacturer’s protocols.

### Assessment of cardiac function and myocardial infarct size

Cardiac function was measured by invasive hemodynamic evaluation methods at 3 h post-reperfusion as previously described [26]. After 24 h of reperfusion, echocardiography was conducted with a VEVO 2100 platform (Visual Sonics, Toronto, Canada). M-mode images were used to obtain left ventricular end-systolic volume (LVESV) and left ventricular end-diastolic volume (LVEDV) measurements and to calculate the percent ejection fraction (%EF). After echocardiographic examination, myocardial infarct size (IS) was assessed by Evans blue/triphenyl tetrazolium chloride (TTC) double staining and calculated as a percent of infarcted area over total area-at-risk (% INF/AAR) as described before [27, 28].

### Determination of serum cardiac troponin I (cTnI) and lactate dehydrogenase

Blood samples were collected from the carotid artery after 3 h of reperfusion period. Serum cardiac troponin I (cTnI) and lactate dehydrogenase (LDH) levels were measured by using specific commercial kits (Nanjing jiancheng Reagents, China). The activities of these two enzymes were presented as nanogram per milliliter (ng/ml) and units per liter (U/L) respectively.

### Quantification of cardiomyocyte apoptosis

After 3 h of reperfusion, terminal deoxynucleotidyl nick-end labeling (TUNEL) assay was used to detect cardiomyocyte apoptosis. TUNEL staining was performed as described previously using in situ cell death detection kits (Roche) [19]. The apoptosis index was expressed as the number of apoptotic cells (TUNEL-positive staining)/the total number of nucleated cells (4′,6-diamino-2-phenylindole staining) ×100%. Myocardial caspase-3 activity was measured to detect cardiomyocyte apoptosis with the use of caspase colorimetric assay kits (Chemicon, Temecula, CA, USA) as we described before [29].

### Quantification of malondialdehyde (MDA) and manganese superoxide dismutase activity (MnSOD)

The MDA levels in myocardial homogenates were determined as described before [30]. MnSOD activities were measured spectrophotometrically by using commercial kits (Beyotime, Jiangsu, China) according to manufacturer’s protocols.

### Statistical analysis

All the data are presented as mean ± standard error of the mean (SEM). The differences of all measured parameters were assessed by One-way ANOVA followed by Bonferroni post hoc test with the use of GraphPad Prism software version 5.0. Probabilities of <0.05 were taken as statistically significant.

## Results

### Characterization of animals

As shown in Table 1, compared with the non-diabetic mice, diabetic animals manifested significantly increased blood glucose and serum TG, indicating that type 2 diabetic model was created in this study. There was a small trend toward decreased serum insulin level and increased body weight that did not reach significance in diabetic mice compared to non-diabetic mice.

**Table 1:** Characterization of animals

Groups	Non-diabetic	DM
Blood glucose (mmol/L)	4.6 ± 0.7	15.8 ± 2.3^**^
Serum insulin (mIU/L)	15.8 ± 2.1	13.2 ± 3.4
Serum TG (mmol/L)	0.46 ± 0.11	1.32 ± 0.18^**^
Body weight (g)	25.3 ± 2.5	28.8 ± 3.1

Values presented are mean ± SEM

*DM* high-fat diet-fed streptozotocin (HFD-STZ) diabetic group, *TG* triacylglycerol

N = 8

** *p* < 0.01 vs. Non-diabetic

### Mdivi-1 inhibited Drp1 translocation to the mitochondria and reduced mitochondrial fission following I/R in diabetic hearts

**Fig. 2:**
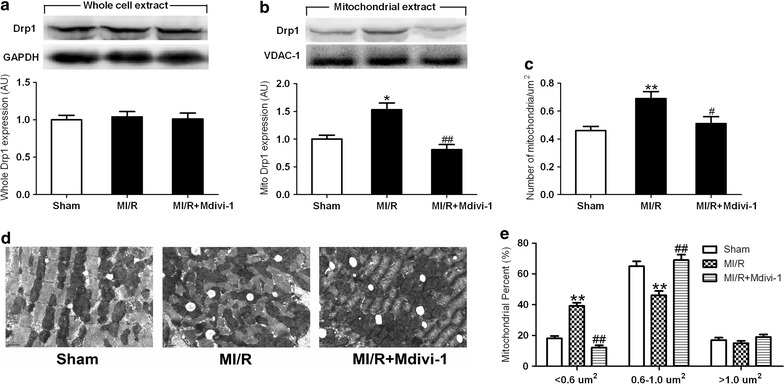
Mdivi-1 inhibited Drp1 translocation to the mitochondria and prevented mitochondrial fission following MI/R in diabetic mice. **a** Total Drp1 expression. **b** Mitochondrial Drp1 expression. **c** Number of mitochondria per μm^2^ in each field of view. **d** Representative transmission electron microscopic images (major finding is **c**, **e**). **e** Percentage of mitochondria that sorted into three size categories based on area. MI/R group means diabetic mice subjected to MI/R and vehicle (dimethyl sulfoxide) administration. Values are mean ± SEM. N = 3–6 hearts for each group. *P < 0.05, **P < 0.01 vs. Sham. ^#^ *P* < 0.05, ^##^ *P* < 0.01 vs. MI/R

As one major regulator of mitochondrial fission, Drp1 typically resides in an inactive form in the cytosol and on activation translocates to the mitochondria. Drp1 will not dislocate from the mitochondria after fragmentation. The method that evaluates Drp1 activity by determining Drp1 protein level in the whole cell and mitochondria has been widely used in recent studies [31, 32]. Here, changes in the total Drp1 expression were not observed (Fig. 2a), while mitochondrial Drp1 expression in diabetic MI/R hearts was significantly increased compared to diabetic sham-operated hearts. By contrast, Mdivi-1 reduced mitochondrial Drp1 expression following MI/R compared to MI/R receiving vehicle administration in diabetic mice (*P* < 0.01, Fig. 2b).

To determine whether changes in mitochondrial morphology were associated with Drp1 activation following MI/R, we isolated and prepared proximal areas to artery occlusion site for transmission electron microscopy. More mitochondria per μm^2^ were observed in I/R hearts compared to sham-operated hearts (*P* < 0.05, Fig. 2c, d). Moreover, there was a significant increase in the percentage of mitochondria smaller than 0.6 μm^2^ in diabetic vehicle-treated MI/R hearts compared to diabetic sham-operated hearts (39.2 ± 2.1 vs. 18.1 ± 1.5% in Sham group, *P* < 0.01, Fig. 2d, e), suggesting enhanced mitochondrial fission. Diabetic MI/R mice treated with Mdivi-1 displayed reduced levels of mitochondrial fission compared to vehicle-treated mice as evidenced by decreased number of mitochondria per μm^2^ and decreased percentage of mitochondria smaller than 0.6 μm^2^ (12.1 ± 1.6 vs. 39.2 ± 2.1% in MI/R group, *P* < 0.01, Fig. 2d, e). There was no significant difference in the percentage of mitochondria bigger than 1 μm^2^ among all the groups. The reason for this may be that elongated mitochondria are more resistant to MI/R injury than middle mitochondria. These findings suggested that Drp1-mediated mitochondrial fission was enhanced in diabetic MI/R hearts, and inhibition of Drp1 reduced mitochondrial fission in diabetic animals following MI/R.

### Drp1 inhibition improved mitochondrial function in diabetic MI/R mice

**Fig. 3:**
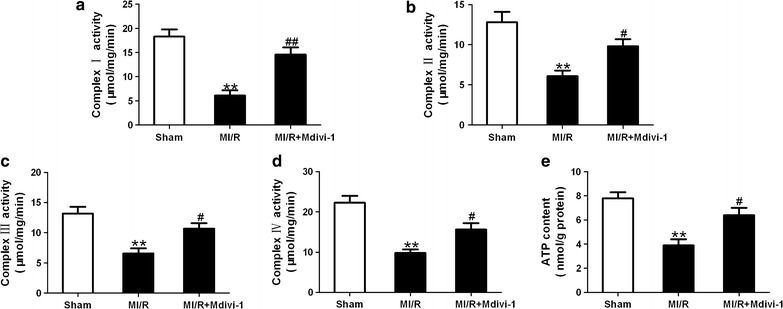
Mdivi-1 improved mitochondrial function in diabetic mice subjected to MI/R. **a** Complex I activity. **b** Complex II activity. **c** Complex III activity. **d** Complex IV activity. **e** ATP content. MI/R group means diabetic mice subjected to MI/R and vehicle (dimethyl sulfoxide) administration. Values are mean ± SEM. N = 8 hearts for each group. **P < 0.01 vs. Sham. ^#^ *P* < 0.05, ^##^ *P* < 0.01 vs. MI/R

It has been suggested that mitochondrial dynamics exerts a pivotal role in controlling mitochondrial function [33]. Here, mitochondrial complex I/II/III/IV activities and ATP content were significantly decreased in MI/R group treated with vehicle compared with sham group (*P* < 0.01, Fig. 3). Mdivi-1 increased mitochondrial complex I/II/III/IV activities and ATP content in I/R myocardium (*P* < 0.05 or *P* < 0.01, Fig. 3), indicating that Drp1 inhibition improved mitochondrial function in diabetic MI/R hearts.

### Drp1 inhibition improved cardiac function in diabetic MI/R mice

**Fig. 4:**
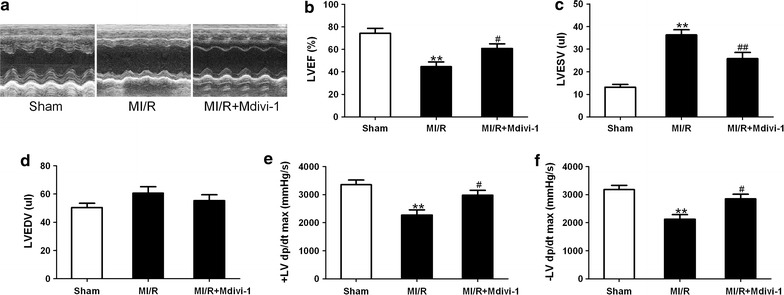
Mdivi-1 improved cardiac function in diabetic mice subjected to MI/R. **a** Representative echocardiography images. **b** Left ventricular ejection fraction (LVEF). **c** Left ventricular end-systolic volume (LVESV). **d** Left ventricular end-diastolic volume (LVEDV). (E and F) ± LV dp/dt max, the instantaneous first derivation of left ventricle pressure. MI/R group means diabetic mice subjected to MI/R and vehicle (dimethyl sulfoxide) administration. Values are mean ± SEM. N = 8 animals for each group. **P < 0.01 vs. Sham. ^#^ *P* < 0.05, ^##^ *P* < 0.01 vs. MI/R

We next sought to explore if Mdivi-1 treatment would improve cardiac function following MI/R in diabetic mice. Echocardiography evaluated at 24 h post-reperfusion revealed that MI/R mice received Mdivi-1 treatment exhibited increased EF and decreased LVESV as compared with the MI/R group treated with vehicle (EF: 60.7 ± 4.1 vs. 44.6 ± 4.2% in MI/R group, *P* < 0.05, Fig. 4b; LVESV: 25.8 ± 2.7 vs. 36.3 ± 2.3 μL in MI/R group, *P* < 0.01, Fig. 4c). No significant differences in LVEDV were observed among all groups. Hemodynamic measurements performed at 3 h post-reperfusion indicated that the ±LV dp/dt max were increased in the MI/R + Mdivi-1 group as compared with the MI/R group treated with vehicle (+LV dp/dt max: 2976 ± 175 vs. 2274 ± 183 mmHg/s in MI/R group, *P* < 0.05, Fig. 4e; −LV dp/dt max: 2846 ± 169 vs. 2124 ± 163 mmHg/s in MI/R group, *P* < 0.05, Fig. 4f). These results suggested that Drp1 inhibition improved cardiac function in diabetic mice subjected to MI/R.

### Drp1 inhibition attenuated MI/R injury in diabetic mice

**Fig. 5:**
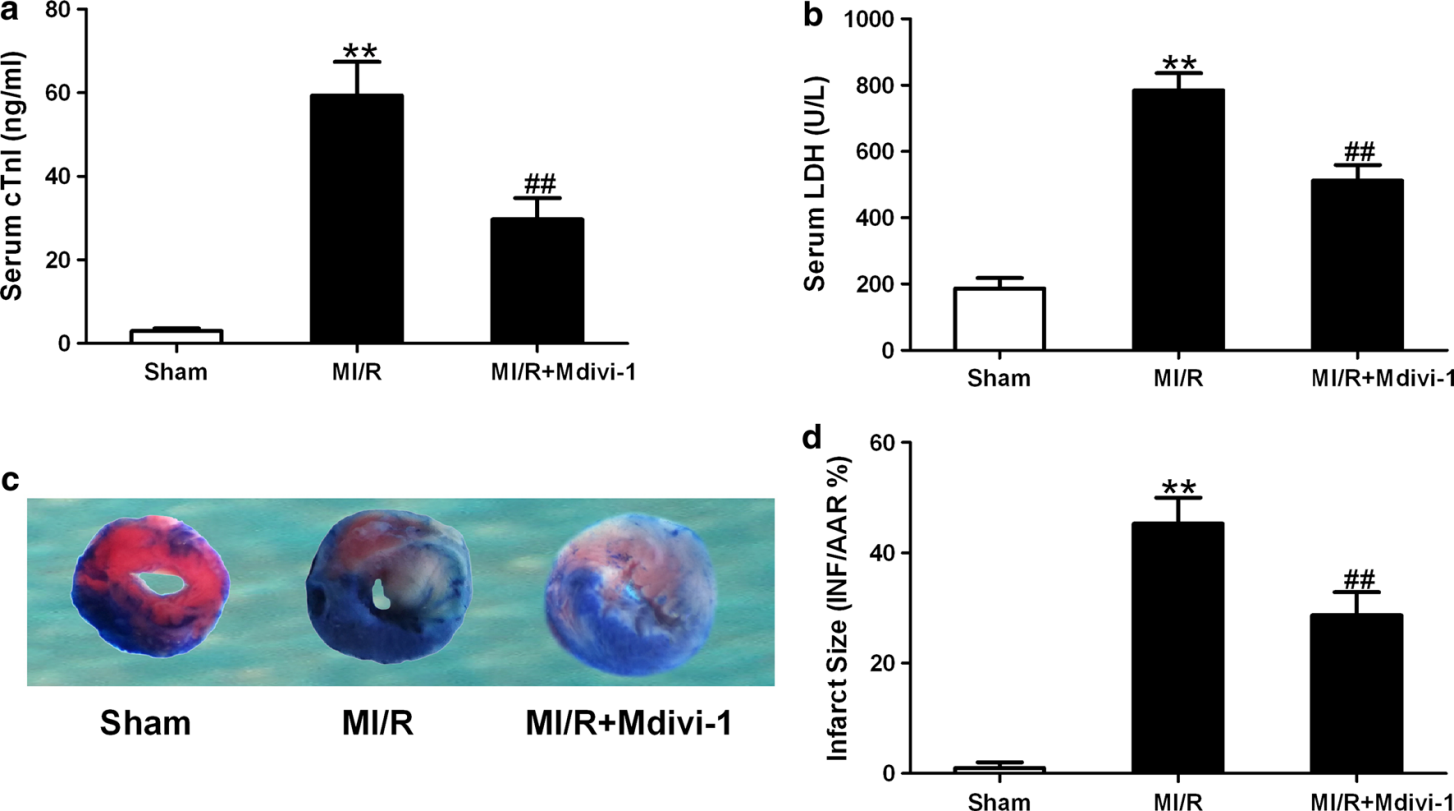
Mdivi-1 reduced myocardial ischemia/reperfusion (MI/R) injury in diabetic mice. **a** Serum levels of cardiac troponin I (cTnI). **b** Serum levels of lactate dehydrogenase (LDH). **c** Representative images of myocardial infarct size stained by Evans blue and TTC. **d** Myocardial infarct size presented as percentage of infarct area (INF)/area at risk (AAR). MI/R group means diabetic mice subjected to MI/R and vehicle (dimethyl sulfoxide) administration. Values are mean ± SEM. N = 8 animals for each group. **P < 0.01 vs. Sham. ^##^ *P* < 0.01 vs. MI/R

To determine whether Drp1 inhibition might reduce MI/R injury, we measured serum cTnI and LDH levels and myocardial infarct size. Compared with sham group, serum cTnI and LDH levels were significantly increased in MI/R group treated with vehicle. Mdivi-1 administration reduced serum cTnI and LDH levels compared with that of MI/R group treated with vehicle (cTnI: 29.7 ± 5.1 vs. 59.3 ± 8.1 ng/mL in MI/R group, *P* < 0.01, Fig. 5a; LDH: 512 ± 47 vs. 784 ± 52 U/L in MI/R group, *P* < 0.01, Fig. 5b). Moreover, the MI/R mice received Mdivi-1 treatment show a significant reduction in myocardial infarct size (28.7 ± 4.2 vs. 45.3 ± 4.7% in MI/R group, Fig. 5c, d, n = 8, *P* < 0.01). The effects of Mdivi-1 on non-diabetic MI/R injury were also assessed in our preliminary study. It was found that inhibition of Drp1 significantly reduced infarct size and serum cardiac troponin I activity in MI/R mice without diabetes (Infarct size: 21.6 ± 2.7 vs. 35.1 ± 3.8% in MI/R without diabetes; cTnI: 18.3 ± 2.4 vs. 39.2 ± 3.6 ng/mL in MI/R without diabetes, n = 5, *P* < 0.05 or *P* < 0.01).

**Fig. 6:**
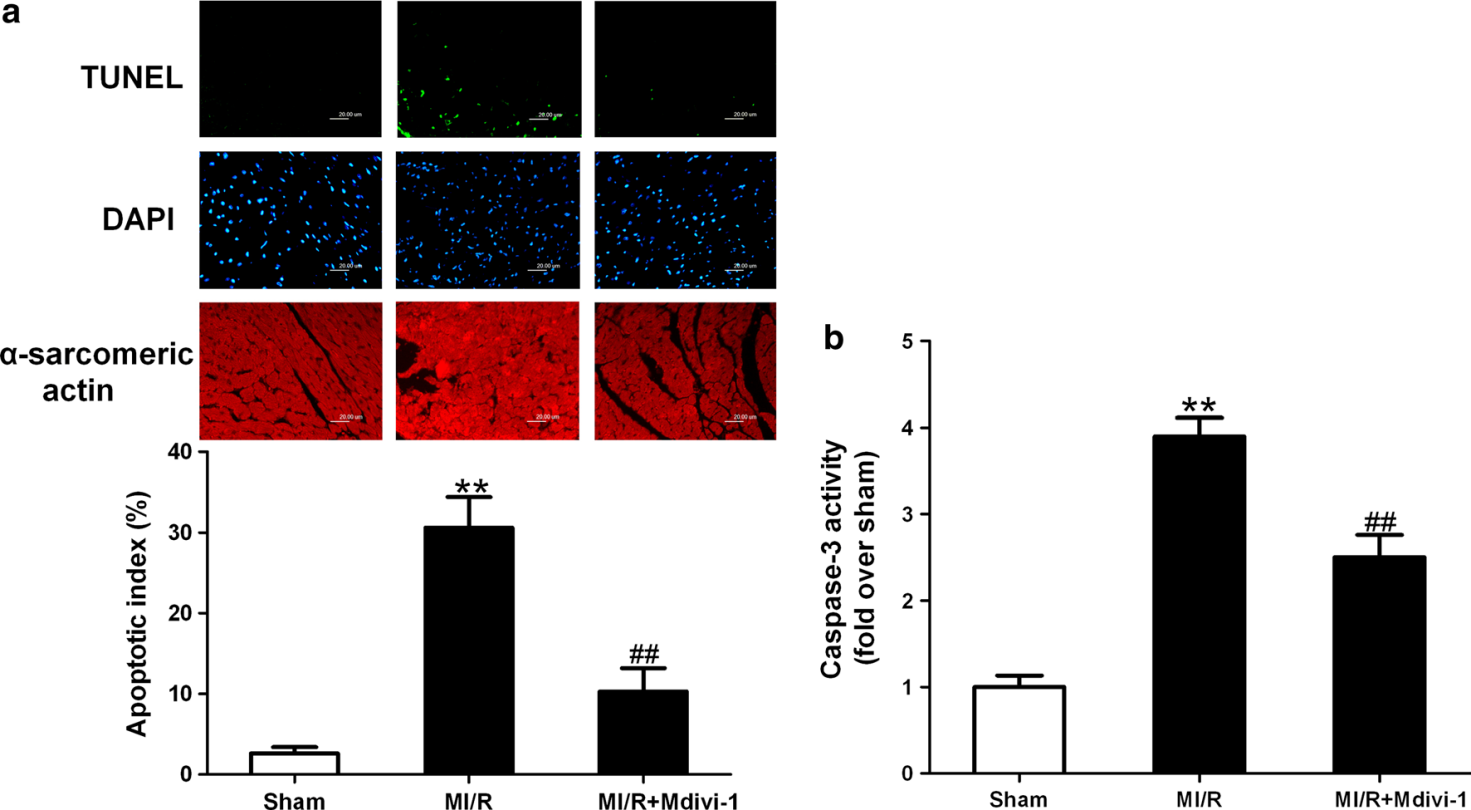
Mdivi-1 suppressed myocardial apoptosis following myocardial ischemia/reperfusion (MI/R) in diabetic mice. **a** *Top* representative terminal deoxynucleotidyl nick-end labeling (TUNEL)-stained and 4’,6-diamino-2-phenylindole (DAPI)-stained photomicrographs. Original magnification ×400. *Bottom* percentage of apoptotic cells (*green fluorescence*)/the total number of nucleated cells (*blue fluorescence*). **b** Myocardial caspase-3 activity (fold over Sham). MI/R group means diabetic mice subjected to MI/R and vehicle (dimethyl sulfoxide) administration. Values are mean ± SEM. N = 8 hearts for each group. **P < 0.01 vs. Sham. ^##^ *P* < 0.01 vs. MI/R

Another major form of cardiomyocyte death following MI/R is apoptosis, we then sought to determine whether Drp1 inhibition could reduce I/R-induced myocardial apoptosis under diabetic conditions. The MI/R mice treated with vehicle showed significantly increased apoptosis index (Fig. 6a) and caspase-3 activity (Fig. 6b) compared with that in sham mice. The MI/R mice received Mdivi-1 administration exhibited suppressed myocardial apoptosis as indicated by decreased apoptosis index (10.3 ± 2.9 vs. 30.6 ± 3.8% in MI/R group, *P* < 0.01, Fig. 6a) and caspase-3 activity (2.5 ± 0.26 vs. 3.9 ± 0.21 in MI/R group, *P* < 0.01, Fig. 6b). These results indicated that Drp1 inhibition alleviated I/R-induced myocardial injury under diabetic conditions in mice, which contributed to the improved recovery of cardiac function following MI/R as above.

### Drp1 inhibition reduced oxidative stress in diabetic MI/R mice

**Fig. 7:**
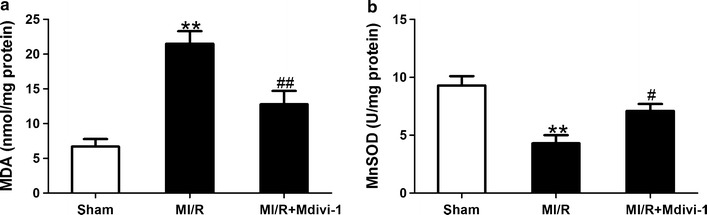
Mdivi-1 suppressed oxidative stress following myocardial ischemia/reperfusion (MI/R) in diabetic mice. **a** The contents of myocardial malondialdehyde (MDA). **b** The activity of mitochondrial manganese superoxide dismutase (MnSOD). MI/R group means diabetic mice subjected to MI/R and vehicle (dimethyl sulfoxide) administration. Values are mean ± SEM. N = 8 hearts for each group. **P < 0.01 vs. Sham. ^#^ *P* < 0.05, ^##^ *P* < 0.01 vs. MI/R

Oxidative damage is a known consequence of MI/R and a likely contributor to cardiac dysfunction. Mitochondrial fission is also a contributor that promotes ROS production [34]. We next sought to determine the effects of Drp1 inhibition on oxidant production following MI/R under diabetic conditions. MDA is a major product of lipid peroxidation and MnSOD is an important antioxidant enzyme protecting mitochondria from oxidative damage, both of which are useful biomarkers for oxidant stress. There was increased MDA production and decreased MnSOD activity in MI/R group treated with vehicle in comparison with the sham group. In contrast, Mdivi-1 administration reduced MDA production and increased MnSOD activity in MI/R mice (MDA: 12.8 ± 1.9 vs. 21.5 ± 1.8 nmol/mg protein in MI/R group, *P* < 0.01, Fig. 7a; MnSOD: 7.1 ± 0.6 vs. 4.3 ± 0.7 U/mg protein in MI/R group, *P* < 0.05, Fig. 7b). These results suggested that Drp1 inhibition reduced I/R-stimulated oxidative injury in diabetic animals.

## Discussion

In this study, we demonstrate for the first time that Drp1-mediated mitochondrial fission is increased following MI/R under diabetic conditions and that its inhibition with Mdivi-1 reduces MI/R injury and improves cardiac function. These findings, coupled with Mdivi-1’s preservation of mitochondrial function and reduction of oxidative stress, indicate an important pathological role of Drp1-mediated mitochondrial fission in diabetic MI/R injury. The findings suggest that Drp1 inhibition may represent a promising novel therapy for diabetic cardiac complications.

It has been demonstrated that mitochondrial dynamics plays an important role in determining mitochondrial morphology and function [35]. However, this important issue has only begun to be addressed in cardiomyocytes during recent years, possibly due to the general perception that spatial constraint of the myofibril architecture in adult ventricular cardiomyocytes may prevent mitochondrial dynamics. Interestingly, the proteins mediating mitochondrial dynamics are abundant in cardiac tissue, suggesting their important roles in cardiac homeostasis [36, 37]. In this study, we showed that I/R induced significant myocardial injury and cardiac dysfunction in diabetic mice, which was accompanied by increased Drp1 activation and mitochondrial fission. Ong et al. have demonstrated that Mdivi-1 administrated 15 min before myocardial ischemia protects the heart against myocardial ischemia/reperfusion (MI/R) injury under non-diabetic conditions [15]. It is important to note that MI/R injury is an acute and unpredictable event. Treatment remedy given at the time of reperfusion reduces MI/R injury would be more meaningful to mimic clinical scenario. In the present study, Mdivi-1 was administrated 15 min before the onset of reperfusion, which may have better translational relevance to the clinical context. Moreover, Mdivi-1 sufficiently prevented mitochondrial fission and reduced MI/R injury in diabetic mice as indicated by decreased serum cTnI/LDH activities and infarct size. To the best of our knowledge, this is the first study not only to find that inhibition of Drp1 reduced MI/R injury under diabetic conditions, but also to reveal that how MI/R injury was reduced (decreased myocardial necrosis and apoptosis and oxidative stress) and how accompanying functional status was changed (improved mitochondrial function and cardiac function) in vivo. Our study extends Ong et al.’s findings and suggests that inhibition of Drp1 may have therapeutic effects for diabetic patients already diagnosed with ischemic heart disease. Targeting other proteins involved in mitochondrial fission may also have cardioprotective effects. Inhibition of the interaction between Drp1 and mitochondrial fission protein 1 (Fis1) using P110 has been showed to reduce myocardial injury following MI/R [38] and may be a promising target for future studies on diabetic cardiac complications.

Mitochondrial fission is associated with most forms of cell death, while mitochondrial fusion protects against apoptosis [39]. Several studies of multiple apoptotic systems revealed that there were causal links between mitochondrial fission and the induction of cell death [10, 40]. During apoptosis, mitochondria become fragmented, which is dependent on the translocation of Drp1 from cytosol to mitochondria [41]. We have found increased Drp1 translocation to the mitochondria was accompanied by enhanced myocardial apoptosis in I/R myocardium of diabetic animals. Moreover, inhibition of Drp1 significantly suppressed myocardial apoptosis as evidenced by decreased TUNEL-positive cells and caspase-3 activity. Our data suggest that Drp1-mediated mitochondrial fission plays a pivotal role in myocardial apoptosis, a major form of cell death following I/R.

Numerous studies have demonstrated the frequent appearance of dysfunctional mitochondria in diabetes and MI/R injury. Mitochondrial functional damage may lead to detrimental consequences on cardiac function and are considered to play an important role in the pathogenesis of cardiovascular disease [42, 43]. In accordance, our study identified that mitochondrial function was significantly impaired by I/R in diabetic hearts, as evidenced by decreased complex I/II/III/IV activities and ATP content. Mdivi-1 effectively rescued electron transport chain (ETC) activities and ATP content. These results indicated that inhibition of mitochondrial fission preserved mitochondrial function in diabetic I/R hearts, which may contribute to the improved recovery of cardiac function as above.

In addition to ATP production, mitochondrial ETC has been recognized as a major source of ROS in cardiomyocytes. It has been demonstrated that mitochondrial dysfunction caused increased ROS generation [44, 45]. In this study, we assessed MDA production and MnSOD activity as surrogate markers of oxidative stress. There was increased MDA production and decreased MnSOD activity following MI/R in diabetic mice. Mdivi-1 treatment increased MnSOD activity and reduced MDA formation in diabetic I/R hearts, indicating that Drp1 inhibition is effective to suppress I/R-induced oxidative stress in diabetic animals. The implied increased ROS following diabetic MI/R injury is generally known and similar to other studies [46–48], but its amelioration with a Drp1 inhibitor following MI/R under diabetic conditions is novel. Since we have provided evidence that Mdivi-1 can rescue the activities of mitochondrial ETC, it is suggested that inhibition of Drp1 may inhibit ROS production directly at the source.

It needs to point out that this study has some limitations. First, we investigated the effects of Drp1 inhibition in a high-fat diet and streptozotocin-induced diabetic model. The efficacy of Mdivi-1 in other diabetic models such as ob/ob mice remains to be studied. Second, Mdivi-1 was used as one pharmacological inhibitor of Drp1 in this study. Our results need to be confirmed with the use of Drp1 conditional knockout mice or Drp1 dominant-negative adenoviruses. Third, our study mainly investigated the effects of Mdivi-1 in vivo. The complex underlying mechanisms mediating the protective effects need to be further explored in vitro.

## Conclusions

The present study demonstrated a critical role of Drp1 in regulating mitochondrial fission and myocardial injury in diabetic mice undergoing MI/R. Pharmacological inhibition of Drp1 prevents mitochondrial fission and reduces MI/R injury in diabetic mice. Our findings suggest that inhibition of Drp1 may be a potential novel therapeutic target for diabetic patients with ischemic heart disease.

## Abbreviations

AAR: area-at-risk
cTnI: cardiac troponin I
Drp1: dynamin-related protein 1
EF: ejection fraction
ETC: electron transport chain
Fis1: fission protein 1
GAPDH: glyceraldehyde-3-phosphate dehydrogenase
HFD: high-fat diet
LDH: lactate dehydrogenase
LVEDV: left ventricular end-diastolic volume
LVESV: left ventricular end-systolic volume
MDA: malondialdehyde
MI/R: myocardial ischemia and reperfusion
MnSOD: manganese superoxide dismutase
PBST: phosphate buffer solution with Tween-20
ROS: reactive oxygen species
STZ: streptozotocin
TTC: triphenyltetrazolium chloride
TUNEL: terminal deoxynucleotidyl nick-end labeling
VDAC1: voltage-dependent anion channel 1

## References

[1] Li H, Liu Z, Wang J, Wong GT, Cheung CW, Zhang L (2013). Susceptibility to myocardial ischemia reperfusion injury at early stage of type 1 diabetes in rats. Cardiovasc Diabetol..

[2] Murase H, Kuno A, Miki T, Tanno M, Yano T, Kouzu H (2015). Inhibition of DPP-4 reduces acute mortality after myocardial infarction with restoration of autophagic response in type 2 diabetic rats. Cardiovasc Diabetol..

[3] Bugger H, Bode C (2015). The vulnerable myocardium. Diabet Cardiomyopathy. Hamostaseologie..

[4] Gross ER, Hsu AK, Gross GJ (2007). Diabetes abolishes morphine-induced cardioprotection via multiple pathways upstream of glycogen synthase kinase-3beta. Diabetes.

[5] Ghaboura N, Tamareille S, Ducluzeau PH, Grimaud L, Loufrani L, Croue A (2011). Diabetes mellitus abrogates erythropoietin-induced cardioprotection against ischemic-reperfusion injury by alteration of the RISK/GSK-3beta signaling. Basic Res Cardiol.

[6] Lejay A, Fang F, John R, Van JA, Barr M, Thaveau F (2016). Ischemia reperfusion injury, ischemic conditioning and diabetes mellitus. J Mol Cell Cardiol.

[7] Bugger H, Abel ED (2010). Mitochondria in the diabetic heart. Cardiovasc Res.

[8] Yan W, Zhang H, Liu P, Wang H, Liu J, Gao C (2013). Impaired mitochondrial biogenesis due to dysfunctional adiponectin-AMPK-PGC-1alpha signaling contributing to increased vulnerability in diabetic heart. Basic Res Cardiol.

[9] Ansley DM, Wang B (2013). Oxidative stress and myocardial injury in the diabetic heart. J Pathol..

[10] Westermann B (2010). Mitochondrial fusion and fission in cell life and death. Nat Rev Mol Cell Biol.

[11] Youle RJ, van der Bliek AM (2012). Mitochondrial fission, fusion, and stress. Science.

[12] Zepeda R, Kuzmicic J, Parra V, Troncoso R, Pennanen C, Riquelme JA (2014). Drp1 loss-of-function reduces cardiomyocyte oxygen dependence protecting the heart from ischemia–reperfusion injury. J Cardiovasc Pharmacol.

[13] Sharp WW, Fang YH, Han M, Zhang HJ, Hong Z, Banathy A (2014). Dynamin-related protein 1 (Drp1)-mediated diastolic dysfunction in myocardial ischemia-reperfusion injury: therapeutic benefits of Drp1 inhibition to reduce mitochondrial fission. Faseb J..

[14] Otera H, Ishihara N, Mihara K (2013). New insights into the function and regulation of mitochondrial fission. Biochim Biophys Acta.

[15] Ong SB, Subrayan S, Lim SY, Yellon DM, Davidson SM, Hausenloy DJ (2010). Inhibiting mitochondrial fission protects the heart against ischemia/reperfusion injury. Circulation.

[16] Yu T, Robotham JL, Yoon Y (2006). Increased production of reactive oxygen species in hyperglycemic conditions requires dynamic change of mitochondrial morphology. Proc Natl Acad Sci USA..

[17] Makino A, Scott BT, Dillmann WH (2010). Mitochondrial fragmentation and superoxide anion production in coronary endothelial cells from a mouse model of type 1 diabetes. Diabetologia.

[18] Gawlowski T, Suarez J, Scott B, Torres-Gonzalez M, Wang H, Schwappacher R (2012). Modulation of dynamin-related protein 1 (DRP1) function by increased O-linked-beta-N-acetylglucosamine modification (O-GlcNAc) in cardiac myocytes. J Biol Chem.

[19] Ding M, Lei J, Han H, Li W, Qu Y, Fu E (2015). SIRT1 protects against myocardial ischemia-reperfusion injury via activating eNOS in diabetic rats. Cardiovasc Diabetol..

[20] Zhang X, Wang Z, Huang Y, Wang J (2011). Effects of chronic administration of alogliptin on the development of diabetes and beta-cell function in high fat diet/streptozotocin diabetic mice. Diabetes Obes Metab.

[21] Shimizu Y, Lambert JP, Nicholson CK, Kim JJ, Wolfson DW, Cho HC (2016). DJ-1 protects the heart against ischemia-reperfusion injury by regulating mitochondrial fission. J Mol Cell Cardiol.

[22] Cui M, Ding H, Chen F, Zhao Y, Yang Q, Dong Q (2016). Mdivi-1 protects against ischemic brain injury via elevating extracellular adenosine in a cAMP/CREB-CD39-dependent manner. Mol Neurobiol.

[23] Ding M, Lei J, Qu Y, Zhang H, Xin W, Ma F (2015). Calorie restriction attenuates monocrotaline-induced pulmonary arterial hypertension in rats. J Cardiovasc Pharmacol.

[24] Marsboom G, Toth PT, Ryan JJ, Hong Z, Wu X, Fang YH (2012). Dynamin-related protein 1-mediated mitochondrial mitotic fission permits hyperproliferation of vascular smooth muscle cells and offers a novel therapeutic target in pulmonary hypertension. Circ Res.

[25] Wang JX, Jiao JQ, Li Q, Long B, Wang K, Liu JP (2011). miR-499 regulates mitochondrial dynamics by targeting calcineurin and dynamin-related protein-1. Nat Med.

[26] Sun D, Shen M, Li J, Li W, Zhang Y, Zhao L (2011). Cardioprotective effects of tanshinone IIA pretreatment via kinin B2 receptor-Akt-GSK-3beta dependent pathway in experimental diabetic cardiomyopathy. Cardiovasc Diabetol..

[27] Adams B, Mapanga RF, Essop MF (2015). Partial inhibition of the ubiquitin-proteasome system ameliorates cardiac dysfunction following ischemia-reperfusion in the presence of high glucose. Cardiovasc Diabetol..

[28] Van der Mieren G, Nevelsteen I, Vanderper A, Oosterlinck W, Flameng W, Herijgers P (2012). Angiotensin-converting enzyme inhibition and food restriction in diabetic mice do not correct the increased sensitivity for ischemia-reperfusion injury. Cardiovasc Diabetol..

[29] Fu F, Tian F, Zhou H, Lv W, Tie R, Ji L (2014). Semen cassiae attenuates myocardial ischemia and reperfusion injury in high-fat diet streptozotocin-induced type 2 diabetic rats. Am J Chin Med.

[30] Xie N, Zhang W, Li J, Liang H, Zhou H, Duan W (2011). alpha-Linolenic acid intake attenuates myocardial ischemia/reperfusion injury through anti-inflammatory and anti-oxidative stress effects in diabetic but not normal rats. Arch Med Res.

[31] Li Y, Wang P, Wei J, Fan R, Zuo Y, Shi M (2015). Inhibition of Drp1 by Mdivi-1 attenuates cerebral ischemic injury via inhibition of the mitochondria-dependent apoptotic pathway after cardiac arrest. Neuroscience.

[32] Sharp WW, Beiser DG, Fang YH, Han M, Piao L, Varughese J (2015). Inhibition of the mitochondrial fission protein dynamin-related protein 1 improves survival in a murine cardiac arrest model. Crit Care Med.

[33] Zhao J, Lendahl U, Nister M (2013). Regulation of mitochondrial dynamics: convergences and divergences between yeast and vertebrates. Cell Mol Life Sci.

[34] Willems PH, Rossignol R, Dieteren CE, Murphy MP, Koopman WJ (2015). Redox homeostasis and mitochondrial dynamics. Cell Metab.

[35] Mishra P, Chan DC (2016). Metabolic regulation of mitochondrial dynamics. J Cell Biol.

[36] Dorn GN, Vega RB, Kelly DP (2015). Mitochondrial biogenesis and dynamics in the developing and diseased heart. Genes Dev.

[37] Dorn GN (2015). Mitochondrial dynamism and heart disease: changing shape and shaping change. EMBO Mol Med..

[38] Disatnik MH, Ferreira JC, Campos JC, Gomes KS, Dourado PM, Qi X (2013). Acute inhibition of excessive mitochondrial fission after myocardial infarction prevents long-term cardiac dysfunction. J Am Heart Assoc..

[39] Karbowski M (2010). Mitochondria on guard: role of mitochondrial fusion and fission in the regulation of apoptosis. Adv Exp Med Biol.

[40] Marin-Garcia J, Akhmedov AT (2016). Mitochondrial dynamics and cell death in heart failure. Heart Fail Rev.

[41] Youle RJ, Karbowski M (2005). Mitochondrial fission in apoptosis. Nat Rev Mol Cell Biol.

[42] Liu Y, Jin J, Qiao S, Lei S, Liao S, Ge ZD (2015). Inhibition of PKCbeta2 overexpression ameliorates myocardial ischaemia/reperfusion injury in diabetic rats via restoring caveolin-3/Akt signaling. Clin Sci (Lond)..

[43] Sun D, Li S, Wu H, Zhang M, Zhang X, Wei L (2015). Oncostatin M (OSM) protects against cardiac ischaemia/reperfusion injury in diabetic mice by regulating apoptosis, mitochondrial biogenesis and insulin sensitivity. J Cell Mol Med.

[44] Bhat AH, Dar KB, Anees S, Zargar MA, Masood A, Sofi MA (2015). Oxidative stress, mitochondrial dysfunction and neurodegenerative diseases; a mechanistic insight. Biomed Pharmacother.

[45] Pei H, Yang Y, Zhao H, Li X, Yang D, Li D (2016). The role of mitochondrial functional proteins in ROS production in ischemic heart diseases. Oxid Med Cell Longev..

[46] Koka S, Das A, Salloum FN, Kukreja RC (2013). Phosphodiesterase-5 inhibitor tadalafil attenuates oxidative stress and protects against myocardial ischemia/reperfusion injury in type 2 diabetic mice. Free Radic Biol Med.

[47] Kain V, Kumar S, Sitasawad SL (2011). Azelnidipine prevents cardiac dysfunction in streptozotocin-diabetic rats by reducing intracellular calcium accumulation, oxidative stress and apoptosis. Cardiovasc Diabetol..

[48] Li H, Bian Y, Zhang N, Guo J, Wang C, Lau WB (2013). Intermedin protects against myocardial ischemia-reperfusion injury in diabetic rats. Cardiovasc Diabetol..

