# Erratum to: Inhibition of dynamin-related protein 1 protects against myocardial ischemia–reperfusion injury in diabetic mice

**DOI:** 10.1186/s12933-017-0540-8

**Published:** 2017-05-04

**Authors:** Mingge Ding, Qianqian Dong, Zhenhua Liu, Zheng Liu, Yinxian Qu, Xing Li, Cong Huo, Xin Jia, Feng Fu, Xiaoming Wang

**Affiliations:** 1 Department of Geriatrics, Xi’an Central Hospital, Xi’an, 710003 China; 2 Department of Geriatrics, Xijing Hospital, Fourth Military Medical University, 15 Changlexi Road, Xi’an, 710032 China; 3 Department of Natural Medicine, School of Pharmacy, Fourth Military Medical University, Xi’an, 710032 China; 4 Department of Physiology, Fourth Military Medical University, 169 Changlexi Road, Xi’an, 710032 China

## Erratum to: Cardiovasc Diabetol (2017) 16:19 DOI 10.1186/s12933-017-0501-2

After publication of the original article [1], it came to the authors’ attention that Zhenhua Liu’s name had been spelled incorrectly in the author list. The author list is correct in this erratum.
